# Deep learning with attention supervision for automated motion artefact detection in quality control of cardiac T1-mapping

**DOI:** 10.1016/j.artmed.2020.101955

**Published:** 2020-11

**Authors:** Qiang Zhang, Evan Hann, Konrad Werys, Cody Wu, Iulia Popescu, Elena Lukaschuk, Ahmet Barutcu, Vanessa M. Ferreira, Stefan K. Piechnik

**Affiliations:** Oxford Centre for Clinical Magnetic Resonance Research, Division of Cardiovascular Medicine, Radcliffe Department of Medicine, University of Oxford, UK

**Keywords:** Cardiac Magnetic Resonance, T1-mapping, Quality Control, Convolutional Neural Network, Attention Mapping, Attention Supervision

## Abstract

Cardiac magnetic resonance quantitative T1-mapping is increasingly used for advanced myocardial tissue characterisation. However, cardiac or respiratory motion can significantly affect the diagnostic utility of T1-maps, and thus motion artefact detection is critical for quality control and clinically-robust T1 measurements. Manual quality control of T1-maps may provide reassurance, but is laborious and prone to error. We present a deep learning approach with attention supervision for automated motion artefact detection in quality control of cardiac T1-mapping. Firstly, we customised a multi-stream Convolutional Neural Network (CNN) image classifier to streamline the process of automatic motion artefact detection. Secondly, we imposed attention supervision to guide the CNN to focus on targeted myocardial segments. Thirdly, when there was disagreement between the human operator and machine, a second human validator reviewed and rescored the cases for adjudication and to identify the source of disagreement. The multi-stream neural networks demonstrated 89.8% agreement, 87.4% ROC-AUC on motion artefact detection with the human operator in the 2568 T1 maps. Trained with additional supervision on attention, agreements and AUC significantly improved to 91.5% and 89.1%, respectively (p < 0.001). Rescoring of disagreed cases by the second human validator revealed that human operator error was the primary cause of disagreement. Deep learning with attention supervision provides a quick and high-quality assurance of clinical images, and outperforms human operators.

## Highlights

• A multi-stream CNN ResNet classifier customised for CMR T1-mapping motion artefact detection.
• An attention supervision module to guide the training of CNN classifier.
• A multiple human observer analysis of the scoring results to adjudicate human and machine performance.

## 1 Introduction

T1-mapping using cardiovascular magnetic resonance (CMR) imaging is a novel approach for myocardial tissue characterisation with increasing utility in cardiac diagnostic imaging. Native and post-contrast T1-mapping offer quantitative, pixel-wise measures to detect changes in myocardial composition. Native T1-mapping reflects signals from the intracellular and extracellular compartments, whilst extracellular volume (ECV) mapping can indirectly quantify changes in the extracellular space, including the myocardium interstitium and coronary vascular compartments [[1], [2], [3], [4], [5]]. T1 and ECV mapping enable the detection of pathologically important processes related to excess water, for instance in oedema and inflammation [[6], [7], [8]], protein deposition [9], and other T1-altering substances such as fat [10], iron [11] and a range of commonly encountered cardiac conditions [12].

T1-mapping and CMR imaging in general are prone to a variety of artefacts, which can affect accurate diagnosis. Respiratory motion, for instance, poses significant challenges in T1 map reconstruction, and has been described as the main source of artefacts in the classic T1-mapping techniques based on the Modified Look-Locker Inversion Recovery (MOLLI) approach [13]. Respiratory motion can lead to incorrect disease classification, particularly in cases where the myocardium is thin, such as healthy females but also in patients with dilated cardiomyopathy (DCM) [14]. Motion correction (MOCO) has been proposed to improve T1-mapping quality [15]; however, deploying MOCO unselectively without motion detection has been shown to introduce new artefacts [16,17]. Mis-registering extra-cardiac tissue (such as blood pool, pericardial effusion, or fat) into the myocardium can also lead to false diagnoses. Thus, quality assessment by human operators of the original or MOCO data remain an essential part of image analysis, but is time-consuming and prone to human error due to subjectivity and fatigue. In contrast, a machine learning approach can automate the motion artefact scoring process, prevent unnecessary MOCO on good quality T1 maps (which can introduce additional errors), and identify poor quality T1 maps for human adjudication in clinically-robust diagnostic applications of T1-mapping.

Deep Convolutional Neural Networks (CNNs) have recently enabled unprecedented breakthroughs in image processing. Advanced CNN architectures such as AlexNet [18], VGG Network [19], Residual Network (ResNet) [20], Inception Network [21] have been developed with continuous improved accuracy and capacity in classification tasks. The utilisation of CNN in CMR image post-processing has become increasingly prevalent due to the time-intensive, laborious nature of manual methods [22,23]. Limited interpretability of CNN decision making has been a chief concern, particularly for clinical applications, to establish trust and confidence and to guide the training process. In CMR image quality control, if the neural networks focus on features outside of the myocardium of interest, such as the chest wall or gastrointestinal (GI) motion in artefact scoring of the left ventricle (LV), this could lead to false positives and over-fitting.

Visualisation techniques in CNN provide a way to reveal the attention focus of the neural networks in decision making. Saliency mapping [24] and attention maps using class activation maps (CAM) [25] and Gradient-weighted CAM (Grad-CAM) [26] have been proposed to make the CNN models more transparent. Recent work on trainable [27,28] and transferable [29] attention mapping techniques enhance the training with additional supervision on the layer activations in natural image recognition. We hypothesise that providing additional supervision on layer activation leads to more efficient and reliable training mechanisms of neural networks on CMR image analysis tasks. This process imitates the procedure of training a human operator by giving additional guidance to the neural networks on where to look in addition to plain classification scores.

In this paper, we present a customised CNN instance for the task of motion artefact detection in CMR T1-mapping. We modify a multi-stream 3D Residual Network (ResNet) for scoring motion, and utilise the Grad-CAM [26] attention map technique to reveal which region in the image contributes to the scoring. With an ultimate aim for clinical applications, beyond observing the attention maps, we further present a method to supervise the neural networks to pay particular attention to myocardial segments, by introducing an attention supervision module and additional cost function. Agreement between scores by machine and human operator are compared to evaluate the performance of the neural network and the effectiveness of the guided attention technique. Cases of disagreement were adjudicated and scored by a second human validator, to analyse whether the error lies with the human operator or the machine.

Novel contributions of this work include a multi-stream CNN ResNet classifier customised for T1-mapping motion artefact detection, an attention supervision module to guide the training of CNN classifier, and a multiple human observer analysis of the scoring results to adjudicate human and machine performance.

## 2 Methods

### 2.1 Cardiac T1-mapping and motion artefact

**Fig. 1:**
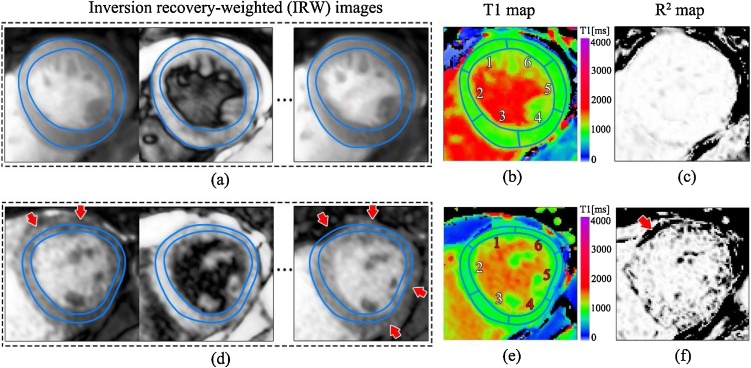
Example cases of T1 maps with good quality breath-hold (top row) and affected by motion artefact (bottom row). Selected inversion recovery weighted (IRW) images (a, d; only 3 of 7 acquired IRW images shown) are used to calculated T1 maps (b, e), with R^2^ maps (c, f; R^2^ - coefficient of explained variance) indicating the quality of T1 fitting. Identical myocardial outlines are overlaid to help identify displacements. Mid-ventricular 6 segments based on the American Heart Association (AHA) model are plotted on T1 maps. In the good quality case, all IRW images (a) have good pixel-to-pixel correspondence, and the R^2^ map (c) shows ‘all white’ across the left ventricular myocardium and cavity. In the bottom case, motion artefact is evident by the misaligned IRW images (d) (arrowed), and the dark bands in R^2^ map (f) at myocardial region (arrowed). In this case, segments 1, 4, 5, 6 (red text) are rejected by an image analyst.

Cardiac T1-mapping, based on Look-Locker method, is calculated by fitting the T1 relaxation curve to a set of inversion recovery-weighted (IRW) images characterised by varying inversion time [30]. The Shortened Modified Look-Locker Inversion Recovery (ShMOLLI) method acquires 7 IRW images within a short 9-heartbeat single breath-hold and reconstructs the T1 map accompanied by the map of coefficient of explained variance R^2^ [14] (Fig. 1). The quality of reconstruction hinges on perfect pixel-to-pixel correspondence between the constituent raw images, so they can be interpreted within the single Bloch equation-based relaxation formula. Due to breathing and poor ECG triggering, this is not always guaranteed. Thus, an R^2^ quality control map (Fig. 1c and f) is necessary to monitor that samples fit well to a mono-exponential T1 relaxation model, as displayed by a uniform white appearance of relevant regions of interest in the R^2^ map (Fig. 1c). Conversely, any displacement in IRW images (Fig. 1d) that shifts tissues with different relaxation into any pixel reduces the applicability of the mono-exponential T1 relaxation equation. This inevitably lowers the coefficient of explainable variation, evident in the R^2^ map as dark bands at the affected areas (Fig. 1f, arrowed). R^2^ is not specific to motion, and its reduction is sensitive to many artefact sources, including off-resonance, fat inclusion, mistriggering and other factors [31,32], usually requiring further investigation by a human operator.

T1 maps were analysed using a dedicated software MC-ROI (MyoCardial Regions Of Interest; programmed by SKP in Interactive Data Language, version 6.1, Exelis Visual Information Solutions, Boulder, Colorado, USA) in accordance to internal guidelines at OCMR [7,33]. A trained human operator was instructed to inspect the T1 map, R^2^ map, and the seven IRW images for scoring each myocardial segment according to American Heart Association (AHA) segmental model [34] for quality and CMR artefacts, including motion, based on the experience in CMR image analysis. Binary per-segment motion artefact labels were then extracted from analysed dataset for training and validating the neural networks for motion detection.

### 2.2 Automated artefact detection with multi-stream CNN image classifier

**Fig. 2:**
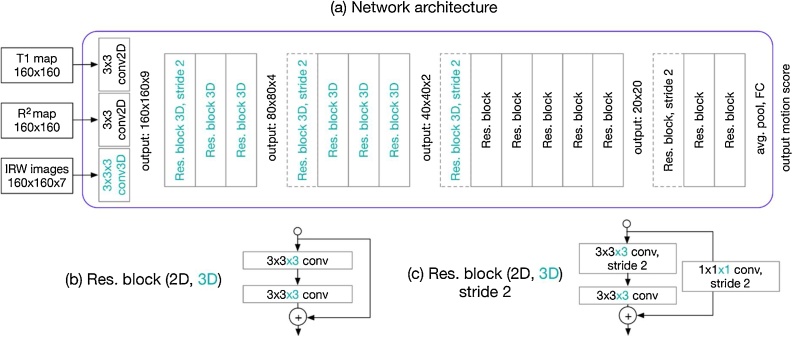
(a) Network structure. The network takes multi-stream input: 7 IRW images, T1 map and R^2^ map (like human operator, Fig. 1), concatenating them after three streams of convolution. 3D convolution is marked in cyan colour. The residual network blocks follow to produce high-level features which are passed to a global average pooling and a fully connected layer for scoring the motion. 3D convolutions are used in the first few blocks. The convolutions are changed to 2D when the third dimension is exhausted. (b) Residual building blocks in the network. The filter size is 3 × 3x3 for 3D and 3 × 3 for 2D. (c) Residual building blocks with a convolutional stride of 2 for down-sampling.

To simulate the human procedures in automating the motion artefact detection, we customised a CNN to integrate the information in the 7 IRW images, as well as the T1 and R2 greyscale maps (Fig. 2). The original IRW images carry direct information on relative movement with added variability introduced by inversion recovery imaging; T1 and R2 maps display strong but non-specific artificial features of motion. All images were cropped centred at the centroid of LV contours based on manual user input with a size of 160 × 160 pixels. A typical convolutional neural network for classification applied convolution and down-sampling on the input images, to learn information ranging from local to more global scales and extract high-level features for decision making. We adopted a 34-layer 3D ResNet [35] architecture and replaced the first convolutional layer with three streams, i.e., two 2D convolution streams on T1 and R2 maps, and a 3D convolution on the stack of 7 IRW images, respectively (Fig. 2a). Kernels in the first convolution layer are of a size of 3 × 3x3 with a stride of 1 instead of 7 × 7x7 as used in the original ResNet due to the smaller image size. The output features were fused into a size of 160 × 160 × 9 and passed to the successive ResNet blocks. Each convolution was followed by batch normalisation and rectified linear unit (ReLU). The feature maps were down-sampled by using a convolutional stride of 2 (Fig. 2) after a few convolutions, to learn features at a more global scale. The convolution was changed to 2D when the third dimension was exhausted due to down-sampling (Fig. 2a). The average pooling was applied following the last convolutional layer to produce compact high-level features, which were passed to the classification layer - a fully connected layer with sigmoid activations to predict the 6 segmental motion scores. Detailed configuration of the network is shown in Table 1 in Appendix A.

### 2.3 Attention visualisation and supervision of CNN classifier

In this section, we describe two attention mapping techniques - saliency map [24] and Grad-CAM [26] - and apply them in motion artefact detection CNN for monitoring whether the machine pays attention to the desired myocardial areas. We further describe the method to guide the machine’s attention towards desired features during the training.

#### 2.3.1 Attention Visualisation

##### 2.3.1.1 Saliency maps

Saliency maps, first introduced in [24], visualise attention by computing the gradient of the output category with respect to input image. This informs how an output category value changes with respect to a small change in input image pixels, therefore the importance of the information the pixel contains in making the decision. The visualisation of these gradients, which are the same shape as the image, should therefore provide some intuition of attention.

Specifically, in our application, given a trained neural network, an input $I\;=\left[I_{T1},\;I_{R2},\;I_{IRW}\right]$ and a target class score l (e.g., motion artefact score by the human observer), the influence weights $W_{T1}$ of pixels from a particular input image (e.g. $I_{T1}$) can be calculated by the derivative $W_{T1}=\partial l/\partial I_{T1}$, as described in [24]. The overall saliency map is then calculated by summarising individual $W_{T1}$, $W_{R2}$ and $W_{raw}$ pixelwise.

##### 2.3.1.2 Grad-CAM

Gradient-Weighted Class activation maps (Grad-CAM) is another way of visualising attention over input [26]. Grad-CAM visualises the nearest convolutional layer to the fully connected layers. The idea is that the last convolutional layer of the CNN contains the spatial information indicating discriminative regions to make classifications. To visualise these parts, Grad-CAM creates a spatial heatmap out of the activations from the last convolutional layer.

Specifically, given a trained neural network with an input, a target class and feature maps $A_{k}\in A,\;k\in [1,2,\ldots ,\;N]$, the neuron importance weights $\alpha_{k}$ of each feature map $A_{k}$ is calculated by global average pooling. The weights $w_{k}$ represent a partial linearisation of the deep network downstream from the last convolutional layer, and captures the ‘importance’ of feature map $A_{k}$ for this target class. The attention map by Grad-CAM $W_{Grad-CAM}$ is then calculated by a combination of forward activation maps $A_{k}$ with weights $w_{k}$, followed by a ReLU to mute the negative values. The final attention map is upsampled to the same size as input images to achieve spatial correspondence.

#### 2.3.2 Attention supervision

**Fig. 3:**
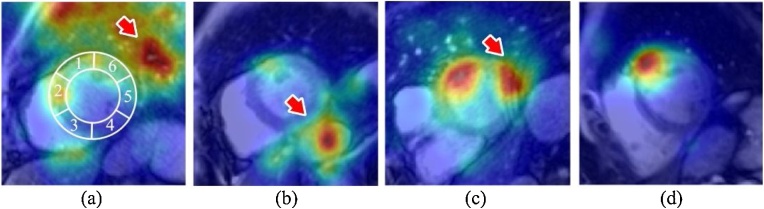
Examples of Grad-CAM attention maps. Distraction of CNN by motion at (a) lung, (b) stomach, and (c) bSSFP banding away from the target segment. (d) Desired attention mapping in which the neural network pays attention to the corresponding myocardial segment. The AHA segment model is overlaid on the first image for reference.

**Fig. 4:**
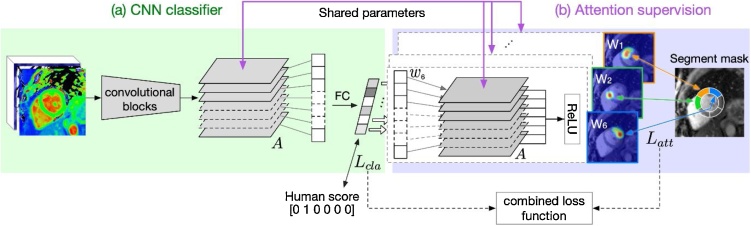
Training a CNN with supervision on attention. (a) The CNN classifier consists of a group of convolutional blocks producing feature maps A, followed by max pooling and fully connected layers to produce per-segment motion scores. (b) For each segmental score $l_{s}$, we compute the neural importance weights $w_{s}$ of feature maps A in the final convolutional layer. The weighted sum of the feature maps is computed, followed by a ReLU operation, to produce the trainable attention map. The respective loss function is imposed on the attention map to encourage attention within the myocardial segment and penalise attention outside. The supervision is passed to the CNN classifier through iterative updates to the parameters of the feature maps A, which are shared between (a) and (b) in this figure. In testing, only the CNN classifier is used, without the attention supervision module.

In CMR, shimming is often applied before image acquisition, a process to address the B0 inhomogeneity of the scanner. While shimming is designed to homogenise the myocardial region of the image, the underlying bSSFP bands can remain close to the myocardial region, in which case related off-resonance effects could cause T1 estimation errors and related mapping artefacts [36] (Fig. 3c). Gastrointestinal and lung displacements are often present in the T1-weighted images. These aspects of motion carry no clinical relevance for myocardial T1 analysis but could introduce distraction for automated motion artefact detection algorithm as revealed by attention mapping (Fig. 3). We propose to feed the CNN with direct additional supervision on attention, by guiding the networks to focus on relevant parts of the image. In this way, the network’s prediction for the task of interest, e.g. quality score of a specific myocardial segment, is based on the relevant areas rather than other parts of the heart or organs. We achieve this process by imposing an attention supervision module (Fig. 4b) on the original CNN classifier (Fig. 4a), which generates an additional term for the cost function as described below.

Following the CNN classifier outputs in section 2.2, to reveal the CNN activation of the last layer activation maps A when predicting the score $l_{s}$ of s˗th myocardial segment (s∈[1, 2, …, 6]), we computed the gradient of the score with respect to the activation maps [27], $\partial l_{s}/\partial A$. The gradients then passed through a global average pooling layer to obtain the neural importance weights $w_{s}$. The neuron weights represented the contribution of layers A in decision making of the artefact score of the specific segment s. We then calculated the weighted combination of the feature maps A using $w_{s}$ as the weights, followed by a ReLU operation to output the attention map $W_{s}$,

$$W_{s}=\text{R}\text{e}\text{L}\text{U}(\sum w_{s}A\;)$$

To guide the machine to pay attention to a specific myocardial segment, a loss function $L_{att}$ is imposed on $W_{s}$ computing the cross entropies between $W_{s}$ and the segment masks $I_{s}$, for all 6 segments,

$$L_{att}=\sum_{s=[1,2,\ldots ,6]}\text{C}\text{r}\text{o}\text{s}\text{s}\text{e}\text{n}\text{t}\text{r}\text{o}\text{p}\text{y}\left(W_{s},I_{s}\right).$$

The attention supervision was imposed on the CNN classifier through shared parameters among the feature maps A in all 6 attention supervision modules and the classifier module (Fig. 4, purple lines).

Classification loss $L_{cl}$ was calculated as the cross-entropy between the segmental scores by the neural networks and the human operator. The final loss function for training the neural network is therefore defined as $L=L_{att}+\alpha L_{cl}$ with α set to one in our experiments.

In the testing phase, the attention supervision module was not used, and ground truth segment masks were not required, while the CNN classifier was kept the same. A standard Grad-CAM module [26] was plugged in to visualise the attention map and compare it with the CNN trained with no attention supervision.

### 2.4 Performance Evaluation

#### 2.4.1 Dataset

We trained and validated the CNN classifiers on 2568 short-axis view basal and mid-ventricular T1 maps from the HCMR study [37], originally contoured and scored by an experienced operator (AB). The data were acquired from multinational centres within the HCMR study using a single T1-mapping method (ShMOLLI). All patients had clinically diagnosed HCM, with unexplained left ventricular hypertrophy (>15 mm), and the dataset contains varied phenotypic manifestations of HCM. The data used for training and validating CNN classifiers (n = 2568) consists of 73% 1.5 T and 27% 3 T T1 maps, and has 321 T1 maps and 1536 segments scored as presence of motion artefact. Motion artefact scores on a six-segment model were extracted from the analysed dataset. We evaluated the performance of automated motion artefact detection with 5-fold cross validation, by randomly partitioning the data into five subsamples, training on three, validating on one and testing on one. The process was repeated five times to obtain the quality scores of the whole data by machine. The performance was assessed by the agreement with human scores, as well as Receiver Operating Characteristics (ROC) curves calculated by thresholding the machine’s classification scores between 0 and 1. The ROC-AUC (Area Under the Curve) were compared using the DeLong test.

To evaluate the performance of motion detection and improvement by the attention supervision on cases in the presence of other CMR artefacts, we also tested the trained CNN classifier on a subset of 163 T1 maps. All these T1 maps were scored to have at least one other artefact besides motion, such as mistriggering, off-resonance, phase irregularities or poor planning.

#### 2.4.2 Implementation specification

We employed on-the-fly augmentation on the training dataset, introducing uniformly distributed random rotation within ±5 degrees and translation within ±10 pixels around the manually annotated centre of LV cavity. The specifications of training CNN were: input size 160 × 160 × 9; batch size 16; initial learning rate 0.001, which was lowered by a factor of 10 at the validation loss plateaus with a patience of 30 epoch. Adam [38] was used as the optimiser. The networks were trained using a NVIDIA TITAN XP GPU. Training was stopped when the validation loss did not decrease for 50 epochs.

#### 2.4.3 Cross-validation of machine and human score disagreements

To analyse the source of disagreement between human and neural network, a subset of T1 maps with at least one disagreed segment was identified for rescoring by a second human validator (CW) who was aware of the disagreement but blinded to the prior scores. Scores of the subset by two human operators and machine were compared for inter-observer variability and identification of machine and human mislabelling.

## 3 Results

### 3.1 Mapping of attention visualisation and supervision effects

**Fig. 5:**
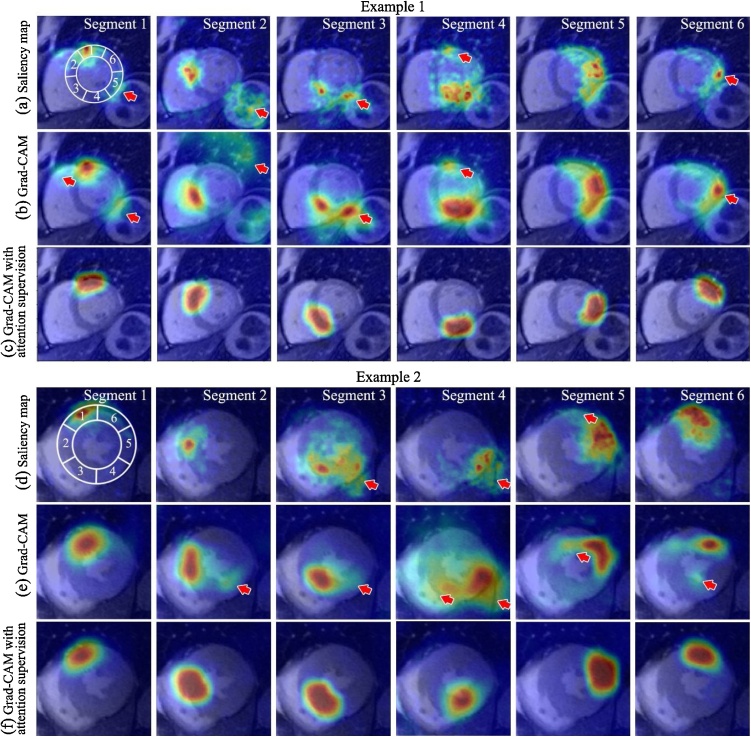
Two examples of attention mapping of the CNN classifier. Traditionally trained CNN classifier for detecting motion shows attention to the relevant myocardial segments, but with distractions (red arrows) and less accuracy, revealed by (a, d) Saliency mapping and (b, e) Grad-CAM techniques. Grad-CAM of CNN classifier trained with the attention supervision module shows accurate focus on the relevant segments, see (c, f). AHA segmental model is overlaid on the first image for reference.

As expected, we found that the CNN classifier trained for predicting segmental motion artefact scores learns to automatically pay attention to the corresponding segmental regions when scoring each of the 6 segments, revealed by saliency maps (Fig. 5a, d) and Grad-CAM techniques (Fig. 5b, e). Saliency maps appeared sharp but noisy as they calculate the pixel-wise derivatives at image resolution. Grad-CAM produced visibly smoother maps due to the low resolution of the last convolutional layer. In the meantime, both visualisation techniques evidenced that the neural networks can be distracted and extend far outside the desired myocardial segments. For example, in (a, b, d, e), segment #2, although the attention map highlighted the septal myocardium, it did not accurately cover the anteroseptal and inferoseptal segments. This was possibly due to the fact that the anteroseptal and inferoseptal motion artefacts often occurred together, making it more difficult for the machine to learn which segment to pay attention to without more specific guidance. In example 1, clear instances of distractions by the gastric motility were seen in all segments, and by the right ventricle in segment #1. In both examples, distractions by other myocardial region were seen in all segments.

In comparison, The CNN classifier with attention supervision pays attention to the desired segment more accurately and specifically (Fig. 5c, f), with no distraction by other myocardial segments, right ventricle or gastrointestinal motion compared to the panels directly above in Fig. 5.

### 3.2 Automatic motion artefact scoring

**Fig. 6:**
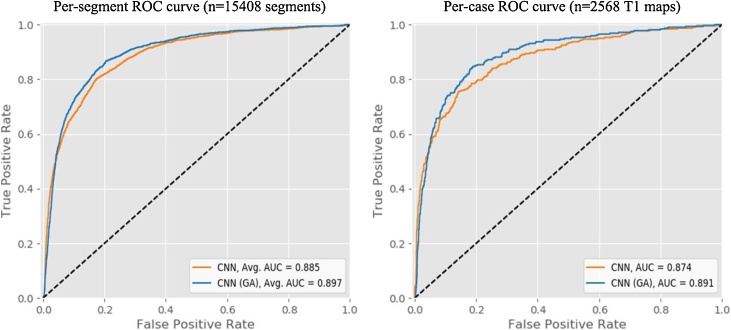
ROC curves for machine performance in the identification of motion artefacts on cardiac T1 maps using a single human operator as the gold standard. Guided attention (GA) technique improves the per-segment (n = 15408) and per-case (n = 2568) ROC-AUC of the CNN classifier in motion detection (p-value = 0.004, <0.001, respectively, DeLong).

The customised ResNet scores the motion artefact with an average 90.7% agreement with the human operator (AB) on all segments, and 89.8% agreement on a whole-image basis (labelled as motion if at least one segment was scored as having motion artefact) (Table 1). Attention supervision improves the scoring accuracy for all segments, their averages and the whole myocardial motion. Attention supervision significantly improved motion artefact scoring performance, as measured by ROC-AUC of the neural network, from 88.5% to 89.7% (per-segment; p = 0.004) and 87.4% to 89.1% (whole-image; p < 0.001) (Fig. 6). We found no statistically significant differences between results on basal and mid-ventricular slices, as provided in Table 1.

**Table 1:** Agreement of CNN classifiers with the human operator.

Segment	CNN			CNN with guided attention		
	Basal slice	Mid-ventricular slice	Basal and mid-ventricular slice	Basal slice	Mid-ventricular slice	Basal and mid-ventricular slice
1 (anterior)	92.6%	91.0%	91.8 ± 0.9%	93.4%	91.0%	**92.1**±0.7%
2 (anteroseptal)	91.3%	89.8%	90.4 ± 1.0%	92.4%	90.8%	**91.5**±0.9%
3 (inferoseptal)	91.3%	90.4%	90.7 ± 0.3%	92.3%	91.6%	**91.9**±0.4%
4 (inferior)	89.7%	90.8%	90.3 ± 0.9%	90.9%	91.5%	**91.2**±0.6%
5 (inferolateral)	90.8%	89.6%	90.1 ± 0.6%	91.8%	91.0%	**91.4**±0.4%
6 (anterolateral)	91.5%	90.3%	91.0 ± 0.8%	92.5%	91.3%	**91.9**±0.7%
Average all segments	91.2%	90.3%	90.7 ± 0.6%	92.2%	91.2%	**91.7**±0.4%
Whole-image basis	89.7%	90.0%	89.8 ± 1.0%	91.5%	91.6%	**91.5**±0.8%

Accuracy of scores by CNN classifiers trained without and with attention supervision technique on 2568 T1 maps from the HCMR dataset, trained and tested human scores as the gold standard. Attention supervision provided significantly better average accuracy for all segments and accuracy on a whole-image basis (p < 0.001).

**Fig. 7:**
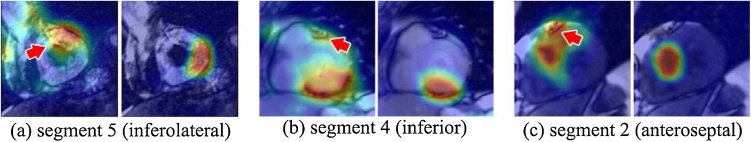
Attention maps on a subset of data with other artefacts. In each panel, LEFT: distractions of CNN classifiers by other artefacts (arrowed). RIGHT: in comparison, CNN classifiers trained with attention supervision focused on the myocardial segments being scored.

On the subset of T1 maps with other artefacts, CNN classifier with no attention supervision module scored the motion with 83.2 ± 4.3% agreement on average with human operators, 7.5% lower than on all T1 maps (90.7%, Table 1). In comparison, CNN classifier with guided attention achieved 90.6 ± 3.0% agreement with human scores, representing a 7.4% improvement over CNN without guided attention, and only 1.1% lower than on all T1 maps (91.7%, Table 1). This demonstrates the robustness of the guided attention module, even in the presence of other artefacts in addition to motion. Examples are given in Fig. 7, which show that CNN classifiers trained without attention supervision were distracted by other artefacts. In comparison, CNN trained with attention supervision focused on the corresponding myocardial segments.

As a supplementary experiment, we tested the stability and sensitivity of the attention supervision module to training data, by replacing the contours in the training data with automated contours generated using a U-net modlifed in our group and trained on in-house datasets for T1-mapping segmentation [40]. The results showed no statistically significant differences between the models trained on manual and automated contours, largely because the automated segmentation neural networks can already achieve human-level performance in accuracy and outperform humans in consistency [[40], [41], [42]]. Two examples of manual and automated contours are provided in Supplementary Fig. 1 for illustration.

### 3.3 Quality check of human and machine scores

**Fig. 8:**
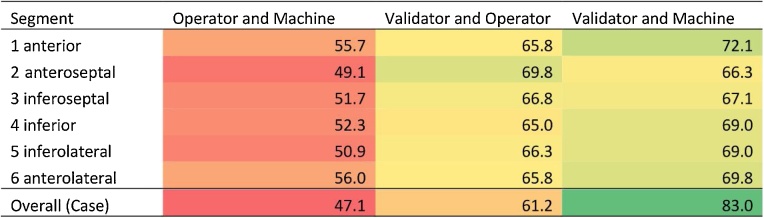
Inter-observer agreement (%) of motion artefacts between the human operator and machine, the human validator and human operator, and human validator and machine on a segmental (AHA model) and case basis. Values are highlighted in colour from the lowest (red) to the highest agreement (green).

The human operator and the network provided identical scores for all six segments in 79% of the T1 maps including basal and mid-ventricular slices. To adjudicate between human or machine mislabelling, the remaining 21% of T1 maps with disagreement of one or more segments were rescored by a second human validator, blinded to prior results but aware of the disagreement. This material represented cases that were difficult to score, or have been mislabelled by either the machine or the first human observer. The overall agreement between the first human operator and the machine was only 47.1% (Fig. 8, column 2). The validator scores agreed more with both the human operator and machine (Fig. 8, columns 3 and 4), with a stark difference showing preference towards automatic machine scores (83.0%), compared to only 61.2% overall agreement between the validator and the human operator. The results revealed that human operator errors were the primary cause of disagreements. The clear errors were possibly due to momentary loss in attention span in performing this long-term repetitive task, for which the Validator had two advantages, with a relatively smaller dataset to score, and advanced knowledge that these were problem cases.

## 4 Discussion

### 4.1 CNN attention visualisation

We have applied visualisation techniques to allow human operators additional insight into the deep learning ‘black box’ in diagnostic medical imaging. Attention heat maps provide a traceable record of the perception process of the machine, offering additional control measures for the accountability required for clinical applications. We showed that traditionally trained networks naturally tend to pay attention to relevant myocardial segments regions when predicting their motion scores. This naturally acquired attention is not uniform and is subject to distractions by other image features outside of the target organ of interest. Attention heat maps helped to explain the source of disagreement in this material, and could provide valuable insights into the accuracy of any machine classification of health and disease for clinical applications in future.

### 4.2 CNN attention supervision

At the cost of providing additional image annotations for the training process, we demonstrated that training the CNN classifier with attention supervision significantly improved the overall classification agreement with the reference human operator across all target structures. CNN classifiers trained without information on segment location have implicitly learnt to locate the corresponding segments for predicting the scores. Attention guidance has made this learning explicit and more specific, and therefore improved the classification accuracy, which may be in future traded for smaller size requirements for the training datasets.

Attention visualisation links this improvement to nearly perfect focus on the myocardial segments in question, and robustness to common extra-cardiac distractors affecting the judgement of the traditionally trained CCN. We also demonstrated that the improvement in any agreement was actually limited by the quality of the human manual annotations; this could affect the analysis of large datasets where the ground truth arises from human labour, which is prone to errors due to fatigue, inattention and inconsistencies.

### 4.3 Quality of human scores in large dataset

Clinical adoption of machine learning for automated diagnostic image analysis has been partly impeded by the long-standing notion that human operators are the gold standard for training automatic algorithms, a belief that current advances in the field seek to challenge. Here, we provide evidence that scores from human operators fall short of a gold standard, due to mislabelling, especially in the context of extremely tedious processing of very large data sets, which require sustained human concentration and consistency over very long periods of time.

We have shown that deep learning trained with noisy labels can provide high accuracy that is comparable to human operators working attentively on a small data sample. This demonstrated that the neural networks can learn the overriding rules and yet avoid reproducing occasional human deviations from these rules. Identifying human mislabelling usually requires a significant amount of work, especially in large-scale studies. Deep learning has the potential to flag potential mislabelling for reinvestigation and correction, and therefore speed up data cleaning.

### 4.4 Limitations and future work

This work limits analysis to the identification of motion artefacts only, rather than addressing all artefacts such as off-resonance, poor planning, phase irregularities, extra-myocardial fat inclusion and other pathology. This is because motion is a prevalent factor affecting a significant proportion of CMR T1 map data and is relatively consistent in detection by human operators, thus subject to the type of analysis performed here. Further work will be required for image assessments to identify rarer and more elusive artefact sources. This paper focused on developing the method and validating it in a single patient cohort dataset, although with a wide range of HCM phenotypes and LV shapes. Current work is underway to extend the training and validation datasets to include other commonly encountered cardiac diseases, for eventual general clinical applications. We also plan in future work to publish or release the source code.

In this work, we used human ground truth to locate and trim the images around the heart. The attention supervision was also trained using manual myocardial segmentation. Automated segmentation algorithms in CMR have been developed and validated, with continual improvements in accuracy and robustness [[41], [42], [43], [44]]. For future work, we aim to integrate the trained motion detection neural networks with robust LV segmentation models for a fully automated pipeline for CMR T1-mapping quality control and processing.

The motion detection CNN classifier, especially when trained with attention supervision, could be used to automatically locate the AHA myocardial segments with a standard Grad-CAM method. This could result in simultaneous automatic segmental localisation and motion artefact scoring, which are two important steps to form an automated pipeline of cardiac T1-mapping analysis. The accuracy of localisation, however, remains to be validated.

This work was validated on short-axis basal and mid-ventricular slices. Apical slices were excluded because the RV and insertion points are often absent in many apical slices; this precluded fair comparisons between machine (who sees slices independently) and human (who sees slices in the context of the whole patient dataset sharing a similar orientation). When scaling up the proposed methods in the future, RV insertion points can be inferred from neighbouring slices and relative slice orientations.

Further work also includes implementing the modules inline on the MR scanner, and applying them offline for a T1-mapping post-processing pipeline. For inline motion detection on the scanner, the radiographers will be notified of any motion at the time of scan, allowing to repeat the breath-holding instruction and data acquisition. In postprocessing, T1 maps with motion artefacts will be detected and motion correction attempted, subject to quality check and potential data exclusion. Impacts of a fully automated pipeline on clinical decision-making and cost-effectiveness need to be assessed in future, with the potential for healthcare cost savings.

## 5 Conclusions

We have demonstrated that the addition of attention maps to deep learning approaches provide useful insights into how neural networks operate, to monitor the training and explain pitfalls. Attention supervision gives additional guidance to neural networks on where to pay attention, leading to significantly improved performance, and exceeded the levels achieved by human operators. We provided evidence that human operators, when processing very large datasets, fall short of a gold standard, and can limit machine learning and performance assessments. Machines can eventually overtake and automate the laborious tasks of image analysis and quality assurance in diagnostic medical imaging.

## Declaration of Competing Interest

SKP has patent authorship rights for U.S. patent 9,285,446 B2. Systems and methods for shortened look locker inversion recovery (ShMOLLI) cardiac gated mapping of T1. Granted March 15, 2016. IP is managed by Oxford University Innovations; the license exclusively transferred to Siemens Healthcare.
