# Supplementary material for: Deep learning with attention supervision for automated motion artefact detection in quality control of cardiac T1-mapping
Source: Artif Intell Med. 2020 Nov;110:101955. doi: 10.1016/j.artmed.2020.101955 (PMC7718111; doi:10.1016/j.artmed.2020.101955)
Supplement: Supplementary file 1 [file mmc1.docx]

# Appendix A

Table 1. Detailed configuration of the neural network based on ResNet.

| layer name | Configuration | output size |
| --- | --- | --- |
| Conv1_2D_T1_map | [3x3, 64], stride 1 | 160x160 |
| Conv1_2D_R^2^_map | [3x3, 64], stride 1 | 160x160 |
| Conv1_3D_IRW_images | $[ 3\times3\times3, 64 ]$, stride 1 | 160x160x7 |
| Concatenate | | 160x160x9 |
| Conv2_x | $\left[ \begin{matrix} 3\times3\times3, 64 \\ 3\times3\times3, 64 \end{matrix} \right]$, stride 2 | 80x80x4 |
|  | $\left[ \begin{matrix} 3\times3\times3, 64 \\ 3\times3\times3, 64 \end{matrix} \right]\times2$, stride 1 |  |
| Conv3_x | $\left[ \begin{matrix} 3\times3\times3, 128 \\ 3\times3\times3, 128 \end{matrix} \right]$, stride 2 | 40x40x2 |
|  | $\left[ \begin{matrix} 3\times3\times3, 128 \\ 3\times3\times3, 128 \end{matrix} \right]\times3$, stride 1 | 40x40x2 |
| Conv4_x | $\left[ \begin{matrix} 3\times3\times3, 256 \\ 3\times3\times3, 256 \end{matrix} \right]$, stride 2 | 20x20x1 |
|  | $\left[ \begin{matrix} 3\times3, 256 \\ 3\times3, 256 \end{matrix} \right]\times5$, stride 1 | 20x20 |
| Conv5_x | $\left[ \begin{matrix} 3\times3, 512 \\ 3\times3, 512 \end{matrix} \right]$, stride 2 | 10x10 |
|  | $\left[ \begin{matrix} 3\times3, 512 \\ 3\times3, 512 \end{matrix} \right]\times2$, stride 1 | 10x10 |
| Fully connected layer | 6-dimension, sigmoid activation | 6x1 |

Residual building blocks are shown in brackets with the number of blocks stacked. Down-sampling is performed by conv2_1, conv3_1, conv4_1, and conv5_1 with a convolutional stride of 2. The network is switched to 2D from conv4_2.

# Appendix B


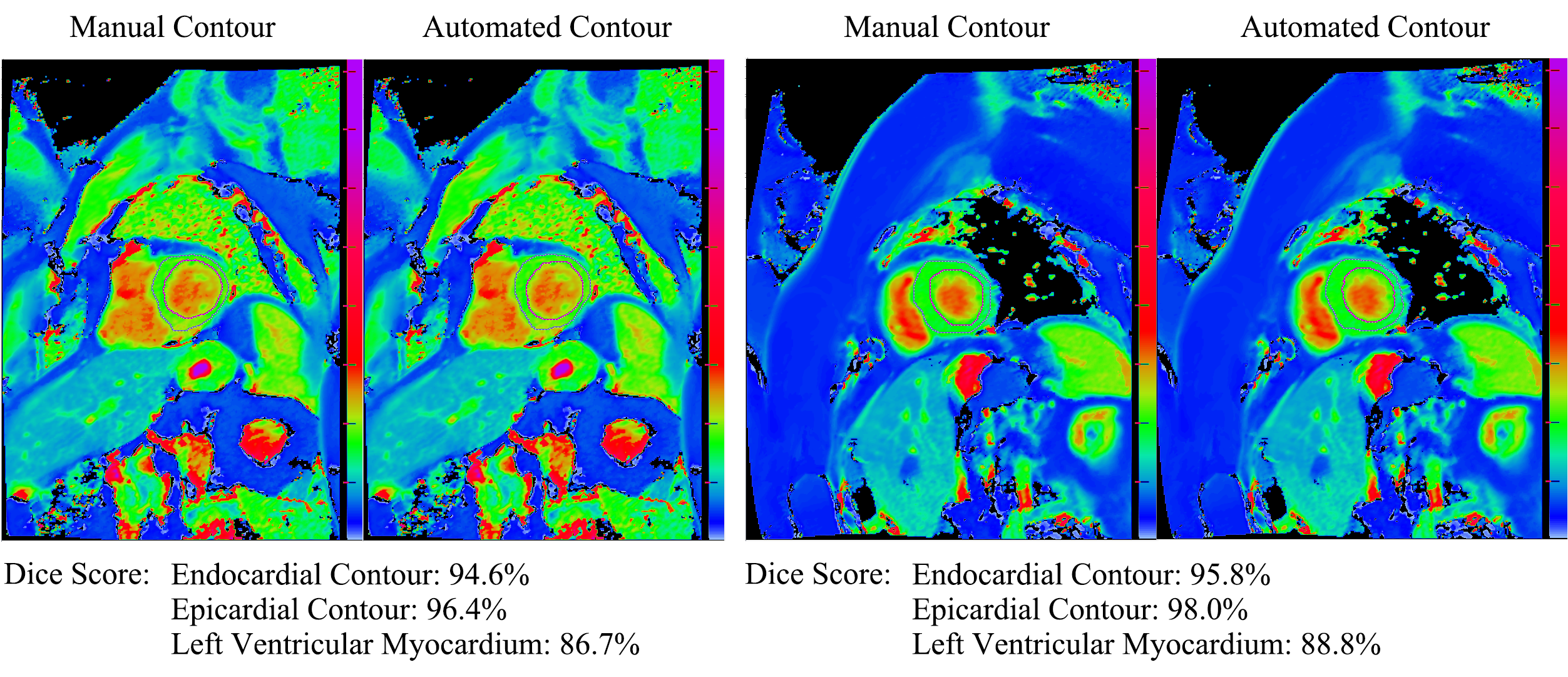


**Supplementary Figure 1:** Examples of manual and automated segmentations and their Dice scores.
